# An Improved Total Variation Minimization Method Using Prior Images and Split-Bregman Method in CT Reconstruction

**DOI:** 10.1155/2016/3094698

**Published:** 2016-08-25

**Authors:** Luzhen Deng, Peng Feng, Mianyi Chen, Peng He, Biao Wei

**Affiliations:** Key Laboratory of Optoelectronics Technology & System, Chongqing University, Ministry of Education, Chongqing 400044, China

## Abstract

Compressive Sensing (CS) theory has great potential for reconstructing Computed Tomography (CT) images from sparse-views projection data and Total Variation- (TV-) based CT reconstruction method is very popular. However, it does not directly incorporate prior images into the reconstruction. To improve the quality of reconstructed images, this paper proposed an improved TV minimization method using prior images and Split-Bregman method in CT reconstruction, which uses prior images to obtain valuable previous information and promote the subsequent imaging process. The images obtained asynchronously were registered via Locally Linear Embedding (LLE). To validate the method, two studies were performed. Numerical simulation using an abdomen phantom has been used to demonstrate that the proposed method enables accurate reconstruction of image objects under sparse projection data. A real dataset was used to further validate the method.

## 1. Introduction

Research on how to reduce Computed Tomography (CT) scanning dose of patients while the image quality is not deteriorated has very important significance not only in theory but also in practical applications [1]. The dose depends on the number of projections, tube voltage, tube current and tube current-exposure time product, X-ray filters, organ shields, and so on. In our study we assume that other factors are fixed during the scanning, except the number of projections. Comparing with traditional CT reconstruction approaches [2–4], algorithms based on Compressive Sensing (CS) [5–10] are more popular with the conditions of incomplete projections. But they still can be improved by bringing in prior images.

Generally, patients are not scanned only once; repeating CT scans contained some same structural information. Normally, the information embedded in previous scanning is called prior knowledge which is valuable for reconstructing better images with low-dose in the following CT scanning [11–15]. The same object can be scanned at different time to monitor the changes of the object. At the first time the object should be scanned with normal views to produce CT images with high quality. Then the subsequent scanning will be carried out under the low-dose circumstance, that is, few-views projections. As the previous normal-dose scans and low-dose scan are not performed simultaneously or even not with the same scanner, the prior images and reconstructed images cannot be utilized directly because of rigid or nonrigid object motion and other differences among these scans. Registration is necessary and how to register them is a huge challenge.

In this paper, we propose an improved Total Variation (TV) minimization method using prior images and Split-Bregman [16] method in CT reconstruction (PISPTV) which tries to take fully advantage of prior images in order to get high-quality CT images with the conditions of incomplete projections. The images obtained asynchronously are registered via Locally Linear Embedding (LLE) and Split-Bregman method is used to solve the optimization problems. We introduce the proposed algorithm in the next section, show the results in the third section, and conclude the paper in the last section.

## 2. Theory and Method

### 2.1. CS-Based CT Reconstruction

For CT reconstruction algorithm based on CS, TV algorithm which is proposed by Sidky and Pan [6] is popular. Briefly, it can be defined as

(1) min⁡ f→TV,s.t. Af→−p→22<σ2,

where $\vec{f}$ is the reconstructed image, *A* is the projection matrix, $\;\vec{p}$ is the projection data, *σ* is permissible error, and the TV of $\vec{f}$, that is, ${\left\|\vec{f}\right\|}_{TV}$, is

(2) $$\begin{array}{c} {\left\|\;\vec{f}\;\right\|}_{TV}=\sum \left|\;\nabla \vec{f}\;\right|=\underset{i,j}{\sum }\sqrt{{\left(\nabla_{x}\vec{f}\right)}^{2}+{\left(\nabla_{y}\vec{f}\right)}^{2}}, \end{array}$$

where $\nabla \vec{f}$ represents gradient operator of an image $\vec{f}$, $\nabla_{x}\vec{f}=f_{i,j}-f_{i-1,j}$, $\nabla_{y}\vec{f}=f_{i,j}-f_{i,j-1}$, *f*_*i*,*j*_ is a pixel value of $\vec{f}$, and *i* and *j* stand for *x*-coordinate and *y*-coordinate, respectively.

### 2.2. Proposed Algorithm

We propose an improved TV minimization method using prior images and Split-Bregman [16] method which can be defined as follows:

(3) min⁡ f→TV+μJPIf→,f→PI,s.t. Af→−p→22<σ2,

where ${\vec{f}}_{PI}$ represents a prior image obtained by a conventional algorithm such as Algebraic Reconstruction Technique (ART) [3] from previous normal-view projections and

(4) $$\begin{array}{c} J_{PI}\left(\;\vec{f},{\vec{f}}_{PI}\;\right)=\underset{t}{\sum }w_{t}{\left\|\;\vec{f}-{\vec{f}}_{PI\left(t\right)}\;\right\|}_{2}^{2}, \end{array}$$

where *t* represents the number of prior images and

(5) $$\begin{array}{c} w_{t}=\exp\left(\;-\frac{{\left\|\vec{f}-{\vec{f}}_{PI\left(t\right)}\right\|}_{2}^{2}}{h^{2}}\;\right), \end{array}$$

where *h* is the parameter to control the sensitivity.

Split-Bregman method is used to solve (3); it contains three iteration steps.

**Step 1 .**

Consider the following:

(6) f→k+1=arg⁡minf⁡ λAf→−p→22+μ∑twtf→−f→PIt22+γd→k−∇f→−b→k22.

**Step 2 .**

Consider the following:

(7) $$\begin{array}{c} {\vec{d}}^{k+1}=\underset{d}{\min}{\left\|\;\vec{d}\;\right\|}_{1}+\gamma {\left\|\;{\vec{d}}^{k}-\nabla {\vec{f}}^{k+1}-{\vec{b}}^{k}\;\right\|}_{2}^{2}. \end{array}$$

**Step 3 .**

(8) $$\begin{array}{c} {\vec{b}}^{k+1}={\vec{b}}^{k}+\left(\;\nabla {\vec{f}}^{k+1}-{\vec{d}}^{k+1}\;\right), \end{array}$$

where *k* is the iteration index of Split-Bregman method, *λ*, *μ*, and *γ* are tuning parameters, and $\vec{d}$ and $\vec{b}$ are intermediate variables.

Equation (6) is solved by the steepest descent method [17] and the derivative of (6) is

(9) g→k,m+1=2λA→mTA→mf→m−pm+2μ∑twtf→m−f→PIt−2γ∇Td→k−∇f→m−b→k,f→m+1=f→m+αdng⌢k,m+1,

where *m* = 2,…, *M* denotes the projection angles, ${\vec{A}}_{m}^{T}$ is the transposition of ${\vec{A}}_{m}$ which is *m*th row vector of $A=({\vec{A}}_{1},{\vec{A}}_{2},\ldots ,{\vec{A}}_{M})$, $\vec{p}=(p_{1},p_{2},\ldots ,p_{M})$, *α* is an appropriate step size, *d*(*n*) is gradient descent scaling parameter of PISPTV, $\overset{⌢}{g}\left[k,m\right]$ is the normalized $\vec{g}[k,m]$, and their relationship is $\overset{⌢}{g}[k,m]={\vec{g}[k,m]/\left\|\vec{g}[k,m]\right\|}_{2}$.

The ART method is used to get initial image of iteration. Equation (7) can be computed as (10) using the shrinkage operator:

(10) $$\begin{array}{c} {\vec{d}}^{k+1}=shrink\left(\;\nabla {\vec{f}}^{k+1}+{\vec{b}}^{k},\frac{1}{\gamma }\;\right). \end{array}$$

### 2.3. Calibration with LLE

Prior images are obtained by ART-TV algorithm from previous normal-view projections. Because the previous normal-dose scans and low-dose scan are not acquired simultaneously or even not scanned using the same scanner, the reconstructed images $\vec{f}$ and ${\vec{f}}_{PI(t)}$ generally are not the same because of rigid or nonrigid object motion and other differences among these scans. In practice we need to register these reconstructed images before further processing, and X-ray CT Geometrical Calibration via Locally Linear Embedding (LLE) which was provided by Chen et al. [18] can be used. In this method, an important step is to calculate the projection matrix which is affected by the geometric parameters; that is,

(11) $$\begin{array}{c} A=A\left(\;\vec{D}\;\right), \end{array}$$

where $\vec{D}$ is a parameter vector containing source-object distance, object-detector distance and detector offset distance, detector tilt angle, projection angle, and so on. The geometric parameters are estimated by the following equation:

(12) D→=arg min p→−p→~s22,s.t. AD→f→=p→s.

$\vec{p}$ is the measured projection data, and ${\tilde{\vec{p}}}_{s}\;\;(s=1,2,\ldots ,S)$ is the corresponding reprojection data during CT reconstruction. *S* is the number of reprojections. The geometric calibration problem is solved by dimensionality reduction via LLE. Specifically, the LLE consists of three steps.

**Step 1 .**

Consider the following:

(13) $$\begin{array}{c} c_{r}={\left(\;\vec{p}-{\tilde{\vec{p}}}_{r}\;\right)}^{T}\left(\;\vec{p}-{\tilde{\vec{p}}}_{r}\;\right). \end{array}$$

**Step 2 .**

Consider the following:

(14) $$\begin{array}{c} \underset{r}{\sum }c_{r}w_{r}=1. \end{array}$$

**Step 3 .**

(15) $$\begin{array}{c} \vec{D}=\overset{R}{\underset{r=1}{\sum }}w_{r}{\tilde{\vec{D}}}_{r}, \end{array}$$

where **C** = (*c*_*r*_) is the local covariance vector, ${\tilde{\vec{p}}}_{r}$ are the *R* nearest reprojection vectors associated with the corresponding *R* vectors of parameters ${\tilde{\vec{D}}}_{r}\;\;({\tilde{\vec{D}}}_{r}=(d_{r1},d_{r2},\ldots ,d_{rR})$ is a densely sampled parametric range), and *w*_*r*_ are weight coefficients. The *R* nearest neighbors are determined by Euclidean distance ${\left\|\vec{p}-{\tilde{\vec{p}}}_{s}\right\|}_{2}^{2}$. The relationship between ${\tilde{\vec{p}}}_{r}$ and $\vec{p}$ is $\vec{p}=\sum_{r=1}^{R}w_{r}{\tilde{\vec{p}}}_{r}$. The weight coefficients here are the same at that for (15). Therefore, the real parameter estimation can be refined by searching for the *R* nearest reprojected projection vectors and updating the parameter vector with the weight coefficients and the corresponding sampled parameters.

### 2.4. Implementation of Proposed Algorithm

**Figure 1:**
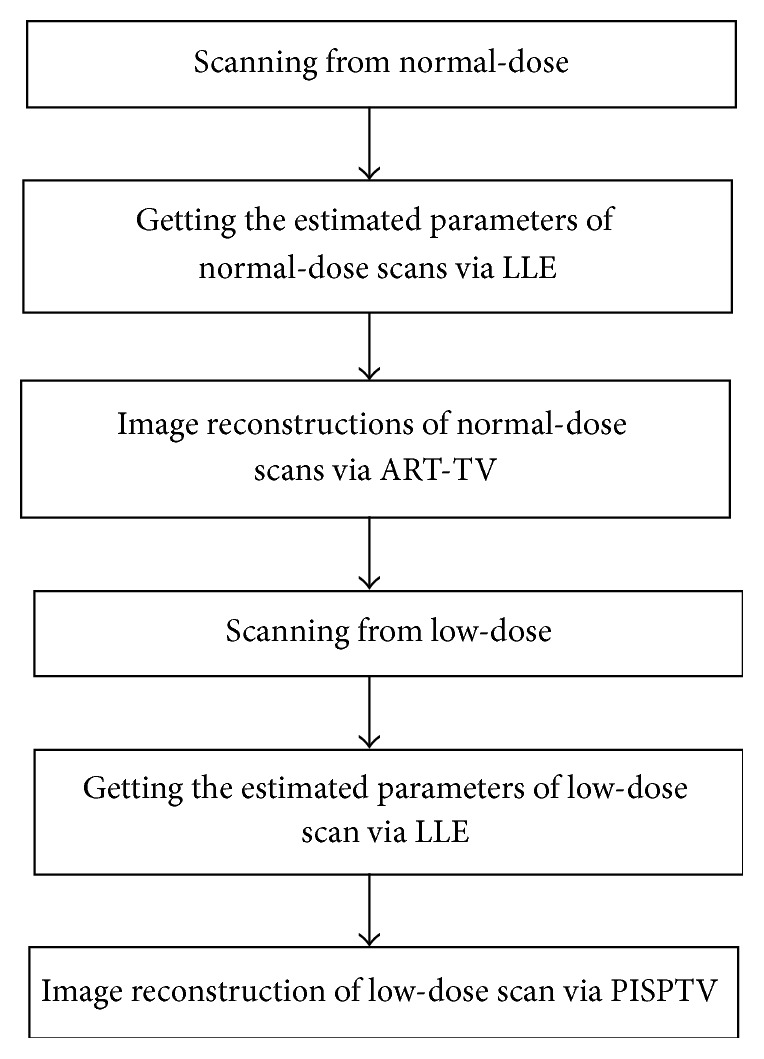
Flowchart for PISPTV reconstruction.

Specifically, our method is flowcharted in Figure 1 and the implementation steps of our proposed algorithm which is shown in Algorithm 1 contain two loops; the outside loop operates ART which is labeled by *n* and the total number of outside iterations is *N*_iter_. The inside loop operates PISPTV which is labeled by *k* and the total number of inside iterations is *K*. ${\vec{f}}_{PI}$ is a prior image obtained by a conventional algorithm such as ART and ART-TV from previous normal-view projections. The prior images are registered via LLE, respectively (the estimated parameter vectors are obtained via LLE before). $\vec{D}$ is an estimated parameter vector obtained by LLE and it is used to register this reconstructed image.

## 3. Simulation and Experiment

To evaluate the performance of our proposed algorithm, the numerical and experimental datasets were used. The ART-TV and our proposed algorithm (PISPTV) were used for comparison. For fairness, both of them were implemented using the Split-Bregman technique. In our study, we selected *α* = 0.15, *λ* = 100, *μ* = 50, and *γ* = 800. Image quality was assessed with the relative Root Mean Square Errors (RMSE) and Structure Similarity (SSIM) [19].

RMSE is the most widely way applied to evaluate image quality, and it is defined as

(16) $$\begin{array}{c} \text{RMSE}=\sqrt{\frac{1}{I\times J}\overset{\;}{\underset{0\leq i<I}{\sum }}\;\overset{\;}{\underset{0\leq j<J}{\sum }}{\left(f_{i,j}-f_{i,j}^{*}\right)}^{2}}, \end{array}$$

where *f*_*i*,*j*_ is the pixel value of original image $\vec{f}$ and *f*_*i*,*j*_^*∗*^ is the pixel value of reconstructed image ${\vec{f}}^{*}$.

SSIM is defined as

(17) $$\begin{array}{c} \text{SSIM}=l{\left(\;\vec{f},{\vec{f}}^{*}\;\right)}^{\delta }\cdot c{\left(\;\vec{f},{\vec{f}}^{*}\;\right)}^{\varsigma }\cdot v{\left(\;\vec{f},{\vec{f}}^{*}\;\right)}^{\eta }, \end{array}$$

where

(18) lf→,f→∗=2f→−×f→−∗+c1f→−2+f→−∗2+c1cf→,f→∗=2σf→σf→∗+c2σf→2+σf→∗2+c2vf→,f→∗=σf→f→∗+c3σf→σf→∗+c3,

where $\overset{-}{\vec{f}}$ and ${\overset{-}{\vec{f}}}^{*}$ are the mean of $\vec{f}$ and ${\vec{f}}^{*}$, respectively. $\sigma_{\vec{f}}^{}$ and $\sigma_{{\vec{f}}^{*}}^{}$ are the variance of $\vec{f}$ and ${\vec{f}}^{*}$, respectively. $\sigma_{\vec{f}{\vec{f}}^{*}}$ is the covariance of $\vec{f}$ and ${\vec{f}}^{*}$. *c*_1_, *c*_2_, and *c*_3_ are small positive constants which are included to avoid instability when denominator in (18) is very close to zero. *δ*, *ς*,  and *η* are used to adjust the weight of luminance $l(\vec{f},{\vec{f}}^{*})$, contrast $c(\vec{f},{\vec{f}}^{*})$, and structures $v(\vec{f},{\vec{f}}^{*})$. In our study, we selected *δ* = *ς* = *η* = 1, *c*_1_ = 2 × 10^−8^, c_2_ = 1 × 10^−8^, and *c*_3_ = c_2_/2 = 5 × 10^−7^.

The value of SSIM is between −1 and 1. When two images are the same, the SSIM between them is 1.

### 3.1. Simulation Study

**Figure 2:**
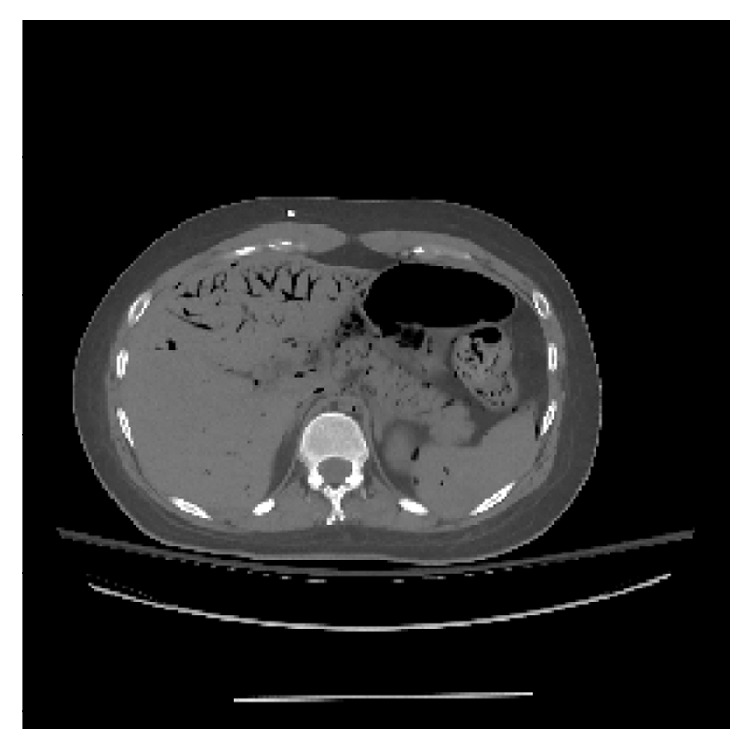
Abdomen phantom.

**Figure 3:**
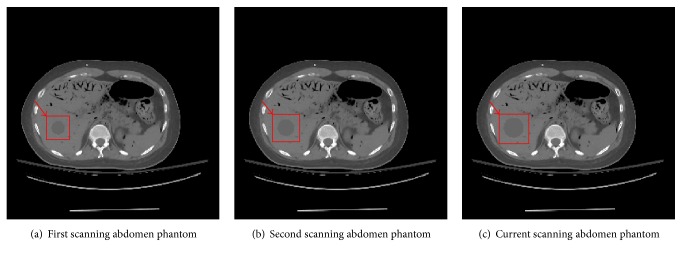
Different scanning abdomen phantoms.

**Figure 4:**
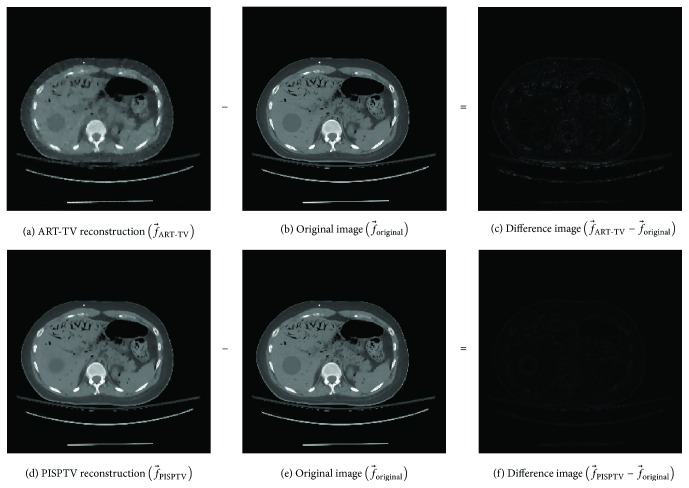
Reconstructed abdomen phantoms for comparison.

In this study, an abdomen phantom as shown in Figure 2 was used. The size of phantom image was 256 × 256. In order to reflect changes of projections in different scan, we added circular patches with different size in the phantom as shown in Figure 3 (their radii were 8 pixels, 10 pixels, and 12 pixels, resp.). The number of prior images was 2. In the first scanning, Figure 3(a) was reconstructed by ART-TV algorithm from 180 projections. In the second scanning, Figure 3(b) was reconstructed by ART-TV algorithm from 90 projections. In the current scanning, Figure 3(c) was reconstructed by PISPTV algorithm from 30 projections. We assumed they were registered in simulation study. The iteration numbers were 50; the reconstructed images are shown in Figure 4.

It can be observed that the image reconstructed by PISPTV is visually much better than that by ART-TV. The differences between Figures 4(a) and 4(d) are clearly identified, which means that PISPTV can produce high-quality image with much less streak artifacts than the ART-TV. Figures 4(c) and 4(f) are the difference images obtained by reconstructed image minus original image. We can see that the difference between ${\vec{f}}_{PISPTV}$ and ${\vec{f}}_{original}$ is much smaller than the difference between ${\vec{f}}_{ART\text{-}TV}$ and ${\vec{f}}_{original}$.

**Figure 5:**
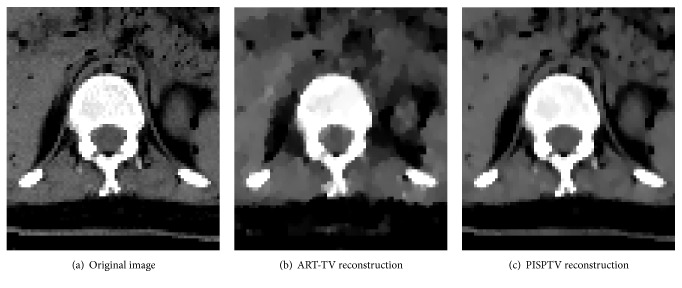
One magnified part of abdomen phantoms for comparison.

Furthermore, we zoom in one part of the reconstructed images as shown in Figure 5. In order to enhance the contrast ratio, all images are shown in the window [0.3, 0.7] while the total grey value is between 0 and 1. We find ${\vec{f}}_{PISPTV}$ contains less artifacts, and the inner distribution near edge is more uniform than ${\vec{f}}_{ART\text{-}TV}$.

Table 1 lists the RMSE and SSIM calculated from reconstructed abdomen phantom with ART-TV and PISPTV. It is obvious that the RMSE of reconstructed image using PISPTV method is much smaller than that of reconstructed image using ART-TV method; the SSIM is much bigger. Thus PISPTV method can reconstruct image with higher quality.

### 3.2. Experimental Study

**Figure 6:**
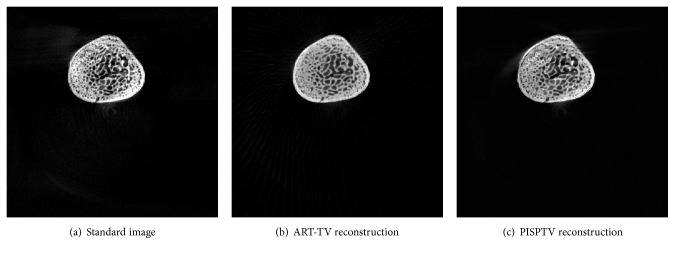
Reconstructed real images for comparison.

In this study, we tested our algorithms on a real dataset from a chip which was acquired by a Micro CT scanner (provided by Nuclear Technology Application Research Center, High Energy Physical Institute, Chinese Academy of Science). The tube was operated at 70 kV and 100 mA. Both the nominal source-object distance and object-detector distance were 38 cm, the number of detector elements was 1024, and the length of detector was 13.0048 cm. Both detector tilt angle and detector offset distance were zero. The calibrated rotation center offset was −0.3847 cm. The number of projection angles was 900 in the angular range [0,2*π*]. These parameters were used to register images. To demonstrate the performance of our proposed approach, we reduced the number of views to 100 which was about 1/9 of original projection number. And as shown in Figure 6(a), we evaluated the reconstructed image using ART-TV with 900-projection data as a standard image. The number of prior images was 3; they were images reconstructed previously by ART-TV algorithm from 900, 450, and 225 projections, respectively. The iteration numbers were 10, and the reconstructed images are shown in Figure 6.

**Figure 7:**
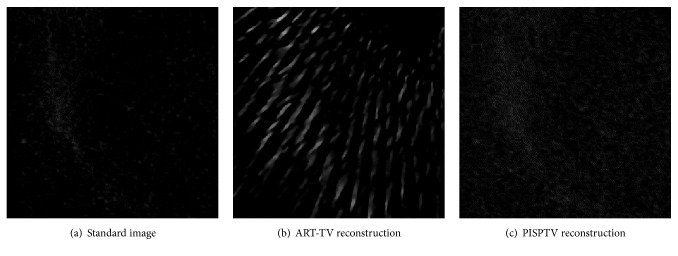
One magnified part of reconstructed real images for comparison.

**Figure 8:**
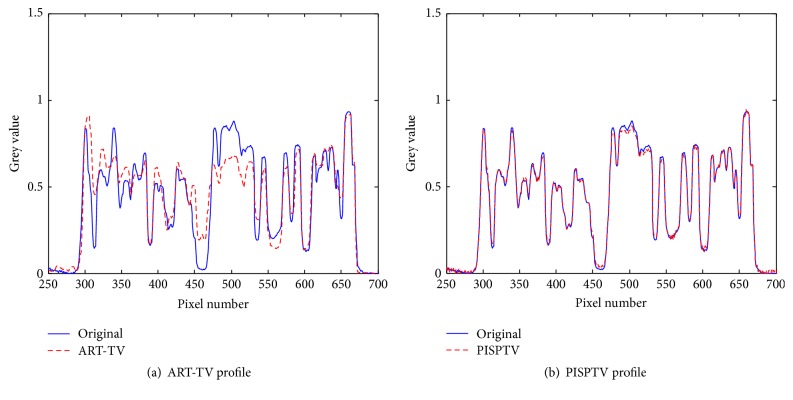
The profile of line 350 in different reconstructed real images.

In Figure 6, it is clear that comparing to ART-TV, PISPTV has better performance. We zoom in one part of the reconstructed images as shown in Figure 7. In order to enhance the contrast ratio and see the artifacts problem, the images are shown in the window [0.01,0.1] while the total grey value is between 0 and 1. We find that the reconstructed image using ART-TV method contains more artifacts. The profiles of line 350 in different reconstructed real images are plotted in Figure 8. We can see that the ART-TV profile fluctuates larger than the PISPTV profile, which means that the PISPTV reconstruction is much closer to standard image. And as shown in Table 1, its RMSE is lower and SSIM is higher. These observations suggest that our method is powerful for sparse CT reconstruction data.

## 4. Conclusion

In conclusion, we propose an improved TV minimization method using prior images and Split-Bregman method which uses prior images to obtain valuable previous information and promote the subsequent imaging process. Split-Bregman method is used to solve the optimal problems. Simulated abdomen phantom and a real dataset are used to validate the method. In the simulation study, different sized circular patches were added to reflect the changes of projections in different scanning. Due to the difficulty of getting real data with these clinical and changes, this kind of comparison will be carried out in further study. For experimental research, X-ray CT Geometrical Calibration via LLE is used to register different reconstructed images. The results demonstrate that the proposed method can reconstruct high-quality images from few-views data and has a potential for reducing the radiation dose in clinical application.

## Figures and Tables

**Algorithm 1:**
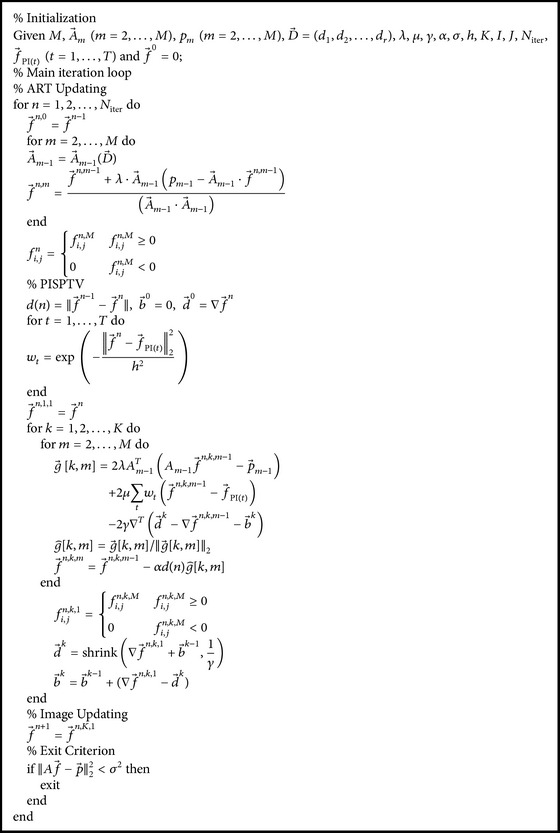
Implementation steps of PISPTV reconstruction.

**Table 1:** RMSE and UQI of reconstruction images.

Methods	Abdomen		Real data	
	ART-TV	PISPTV	ART-TV	PISPTV
RMSE	0.0303	0.0106	0.0414	0.0107
SSIM	0.9877	0.9985	0.9696	0.9971
